# Resistive Switching in Electrodeposited Prussian Blue Layers

**DOI:** 10.3390/ma13245618

**Published:** 2020-12-09

**Authors:** Lindiomar Borges Avila, Christian K. Müller, Dirk Hildebrand, Fabrício L. Faita, Bruna F. Baggio, Cristiani C. Plá Cid, André A. Pasa

**Affiliations:** 1 Laboratório de Filmes Finos e Superfícies, Departamento de Física, Universidade Federal de Santa Catarina, 88040-900 Florianópolis, Brazil; lindiomarbaj@gmail.com (L.B.A.); bfbaggio@gmail.com (B.F.B.); cristiani.campos@ufsc.br (C.C.P.C.); 2 Faculty of Physical Engineering/Computer Sciences, University of Applied Sciences Zwickau, 08056 Zwickau, Germany; Dirk.Hildebrand@fh-zwickau.de; 3 Instituto de Física, Universidade Federal do Rio Grande do Sul, Caixa Postal 15051, 91501-970 Porto Alegre, Brazil; fabriciofaita@gmail.com

**Keywords:** electrodeposition, Prussian blue, resistive switching

## Abstract

Prussian blue (PB) layers were electrodeposited for the fabrication of Au/PB/Ag stacks to study the resistive switching effect. The PB layers were characterized by different techniques to prove the homogeneity, composition, and structure. Electrical measurements confirmed the bipolar switching behavior with at least 3 orders of magnitude in current and the effect persisting for the 200 cycles tested. The low resistance state follows the ohmic conduction with an activation energy of 0.2 eV.

## 1. Introduction

In general, the continuous evolution and improvement of electronic devices have led to new emerging challenges. Many alternatives have been proposed over the years to improve technology, especially in the electronic field. The sizing of the device dimensions was one of those alternatives that made it possible to successfully comply with Moore’s law [1]. However, as the scale for miniaturization increased for electronic devices, different problems related to physical limitations appeared [2,3]. To solve them, the field of material science and device structures, and other physical phenomena were studied. In this sense, a new effect, known as resistive switching (RS), has emerged as an alternative physical mechanism to be implemented in many different applications [4,5]. Devices in which the RS effect occurs are known as memristive devices [6].

Memristors are solid–state-devices where the resistance can be controlled by voltage, retaining the resistance value without power consumption, and that can be used as storage devices [7,8,9]. With the application of an external voltage, the memristor can be switched back and forth between a low resistance state (LRS) and a high resistance state (HRS). Depending upon the polarity of the applied voltage, resistive switching phenomena can be classified as either unipolar or bipolar. Unipolar switching depends on the amplitude of the applied voltage, but not on the polarity of the applied voltage, whereas for bipolar switching, different polarities are used to switch between LRS and HRS (one polarity to switch from LRS to HRS, and the opposite polarity to switch back into LRS). For many systems, the switching mechanism can be ascribed to the conduction filament formation, where the device achieves the LRS, and filament rupture, where the device switches back to the HRS [10]. The theoretical modeling of memristors was presented in 1970 [11], and in 2008 the resistive switching (RS) effect was observed in the laboratory [12]. Currently, several companies and research teams are working to develop these devices for commercial applications, from neuromorphic computing to nonvolatile memories [5,10].

PB is the hexacyanoferrate (II) compound of iron (III) with a cubic face-centered structure, with Fe^3+^ ions coordinated by nitrogen atoms and Fe^2+^ ions coordinated by carbon atoms, resulting in the bonding chain ${\mathrm{Fe}}^{+3}-N\equiv C-{\mathrm{Fe}}^{+2}-C\equiv N-{\mathrm{Fe}}^{+3}$ [13]. When replacing iron ions with other metals such as Co or Mn, a family called Prussian blue analogs is created [14]. Due to the physical and chemical properties of Prussian blue, the study of this material and its analogs has increasingly attracted the attention of the scientific-technological community due to its promising applications in electrochemical sensing, electrochromic devices, batteries, supercapacitors, and magnetic/ photomagnetic systems [15,16,17,18,19]. The growth of PB films has been successfully shown by electrodeposition [20], liquid phase epitaxy [21], and spin coating [22]. Among them, electrodeposition is a very effective method for the growth of PB thin films on a solid substrate due to its high reproducibility, low cost, and ease of controlling deposition parameters [20].

Nevertheless, electrical property characterization of PB films and control of morphology during synthesis are rarely investigated. Conductivity switching in bulk FeCo PB analogs was reported by Sato et al. [23] when varying the temperature and applying high voltages (several hundred volts). However, to our knowledge, resistive switching in PB films has not been studied yet.

In this work, we report the structural properties of electrodeposited PB films with controlled cube morphology. Moreover, the electrical properties associated with the RS phenomenon in electrodeposited thin films of PB are studied. We have observed the bipolar switching mechanism due to different switching polarities between LRS and HRS, high reproducibility of the switching behavior, and a switching ratio of about three orders of magnitude.

## 2. Materials and Methods

### 2.1. Sample Preparation

The electrochemical deposition of the PB films was performed in potentiostatic mode at room temperature by using Au/Cr/Si substrates as working electrodes, a Pt foil as a counter electrode, and a saturated calomel electrode (SCE) as reference. All the voltages in the text refer to this reference electrode. The working electrodes were prepared by evaporating 50 nm Au on 5 nm Cr on (100) Si substrates with a size of 1 cm × 1 cm each at a base pressure of $10^{-5}$ Pa. The deposition of PB occurred in a circular area of 0.5 cm^2^ defined by a mask of adhesive tape on the surface of the working electrode. As electrolyte for the electrochemical synthesis it was used a solution containing 0.25 mM K_3_Fe(CN)_6_, 0.25 mM FeCl_3_, 1.0 M KCl and 5.0 mM HCl at pH 2.2 [24]. The formation of PB layers was obtained by applying a constant potential of 0.3 V and limiting the electrodeposited charge to 30 mC, using an Ivium electrochemical workstation (Ivium CompactStat, Eindhoven, The Netherlands).

### 2.2. Sample Analysis

The morphology of the PB films was investigated with a TESCAN field emission scanning electron microscope (FEG-SEM, TESCAN Amber, Brünn, Czech Republic) at 2 kV. The SEM was equipped with a focus ion beam (FIB) technique allowing to perform a deeper cross-sectional analysis of the PB films. Therefore, a FIB lamella was prepared and analyzed at 30 kV. In addition, the PB distribution and homogeneity were studied by Raman imaging with SEM (RISE) microscopy using a Raman spectrometer (WITec RISE, Ulm, Germany) in combination with an SEM (Zeiss Sigma 300, Oberkochen, Germany). Raman spectra were obtained by using a laser excitation wavelength of 532 nm at a power of 1 mW. The spectra were taken from a PB film area of 15 µm × 15 µm (100 × 100 pixels, 0.1 s integration time per pixel). In parallel, the surface morphology of the same surface region was imaged by SEM operating at 1 kV.

The chemical analysis of the PB films by X-ray photoelectron spectroscopy (XPS) was carried out with a Thermo Scientific K-Alpha using a monochromatic Al Kα source and a spot size of 400 µm. The XPS system was equipped with an Ar-ion-sputtering source for depth analysis of the films. After recording the data, the XPS spectra were analyzed with Casa XPS software version 2.3.23. To compare with XPS data, we have also used energy-dispersive spectroscopy (EDS) measurements using the FEG-SEM microscope described above.

For current versus voltage measurements at room and low temperatures, an Agilent U2722A power system, a Keithley 2400 power meter, and a 10 K Janis CCR Cryostat were used. The 2-point probe method was used with one contact on top of the PB layers (silver glue) and the Au film at the bottom as the second one.

## 3. Results and Discussion

**Figure 1:**
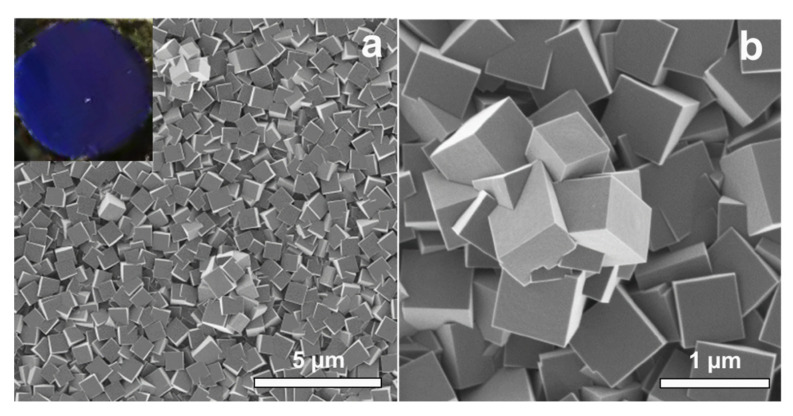
Field emission scanning electron microscope (FEG-SEM) images of electrodeposited Prussian blue (PB) films grown at 0.3 V: (**a**) surface overview, photograph image of the film (inset), (**b**) detailed view of the film surface.

Figure 1 shows typical SEM images of the surface of PB films grown at 0.3 V with a charge of 30 mC. The films showed compact structures with a homogeneous distribution of grains throughout the whole substrate (Figure 1a and inset). The surface morphology of the PB films was predominantly cubic, whereas the edge length of a single cube was less than 1 µm (see Figure 1a,b). Additionally, small amounts of clusters, consisting of several cubes were found on the surface. The preferential growth direction of the cubes was [100] in agreement with previous results [20].

**Figure 2:**
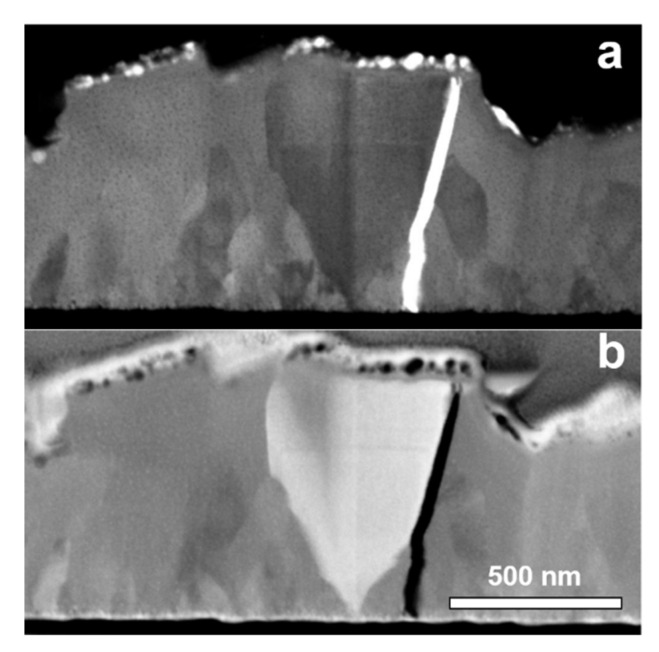
Scanning transmission electron microscopy images taken from the focus ion beam (FIB) lamella of the PB films: (**a**) bright-field image, (**b**) dark-field image.

Figure 2 presents the cross-sectional images of the PB sample prepared with FIB observed in the STEM (scanning transmission electron microscopy) mode. From the images, an average film thickness of 600 nm can be determined. The morphology with grains is clearly visible along the whole layer and with some cubes that have grown from the Au/PB interface up to the PB surface (see Figure 2a,b). However, the grains near the bottom are narrower in size than the grains at the top. Such a grain distribution and the appearance of cube morphology typically occur by Volmer–Weber growth mode, where three-dimensional islands are formed on the surface and grow until they coalesce into a continuous film. Larger grains are more energetically favorable than smaller grains due to Oswald’s ripening and, consequently, dominate the morphology in the upper layers. Additionally, the growth direction of an individual cube can slightly vary from the [100] direction (orientation of the substrate), resulting in the cubes morphology, as seen in Figure 1. Figure 2 also shows a crack that is observed along the thickness of the layer, probably originated due to stress in the film or during the preparation of the lamella.

**Figure 3:**
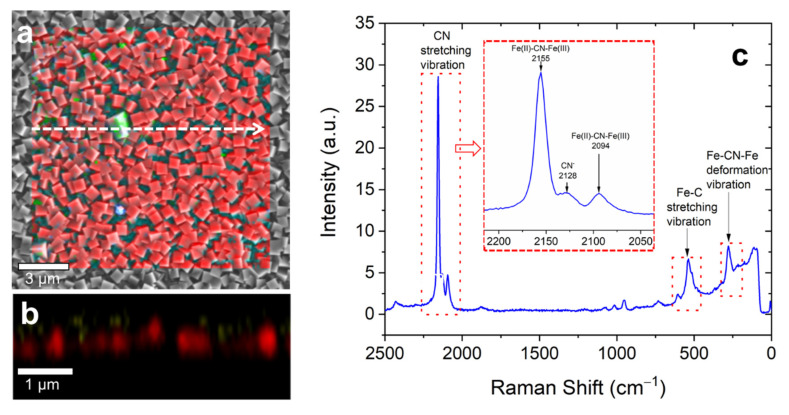
Raman imaging and spectroscopy of thin PB films electrodeposited with 30 mC at 0.3 V: (**a**) Raman area scan with an area size of 15 × 15 µm^2^ (red color) combined with the SEM image of the same sample area. (**b**) Raman depth scan along the marked white arrow in the image (**a**). (**c**) Typical Raman spectrum corresponding to the red-colored regions of images (**a**) and (**b**). The inset shows the region of the CN-stretching vibration in detail. The Raman spectra were recorded at a laser excitation wavelength of 532 nm and a power of 1 mW.

Raman spectroscopy, in combination with electron microscopy, can give useful information about sample materials, material distribution, and structure. Figure 3 summarizes the results obtained by Raman spectroscopy in combination with SEM on our PB films. Raman mappings of the surface (Figure 3a) and in-depth (Figure 3b) prove a homogeneous distribution of PB on both the surface and in-depth. The corresponding Raman spectrum (see Figure 3c) shows the presence of PB, and no significant contaminations from other materials can be found. The Raman spectrum in Figure 3c exhibits the CN stretching bands between 2200 and 2070 cm^−1^, related to the PB cyanide ligands, that are coordinated to iron ions with different oxidation states (Fe^III^−CN−Fe^II^). The main peak at 2155 cm^−1^ refers to the 1 A_g_ ν(CN) stretching vibration of the (Fe(II), Fe(III)) vibrational state [25]. This peak shows a weak shoulder towards lower wavenumbers at 2128 cm^−1^, characteristic for CN^−^ ions [26]. This shoulder can be a spectral signature for different forms of PB, namely sPB (soluble) and iPB (insoluble). It was reported by Moretti and Gervais [27] that the shoulder appears in the case of sPB and not for iPB. Probably, the presence of this weak shoulder at 2128 cm^−1^ in our samples can be an indication of both phases, i.e., the electrodeposited layer is a mixture of iPB and sPB. The second peak appears at 2094 cm^−1^ and refers to the E_g_ mode of the ν(CN) stretching vibration of the [Fe(II), Fe(III)] state [28]. Other characteristic peaks of PB can be found in the lower part of the spectrum. A higher spectral window between 450 and 620 cm^−1^ can be assigned to the Fe–C stretching vibrations of the lattice and appearing with a major peak at 537 cm^−1^ and a second one at 605 cm^−1^ and a lower spectral window (200–350 cm^−1^) with a strong peak at 276 cm^−1^ represent the Fe–CN–Fe bond deformation vibrations [29].

Generally, the observed Raman peaks are relatively broad, with an FWHM of ~10 cm^−1^. Line broadening of the peaks is often discussed in terms of strain [30]. Indeed, cracks observed by SEM (see Figure 2b) can be an indication for strain in our samples resulting in Raman peak broadening.

**Figure 4:**
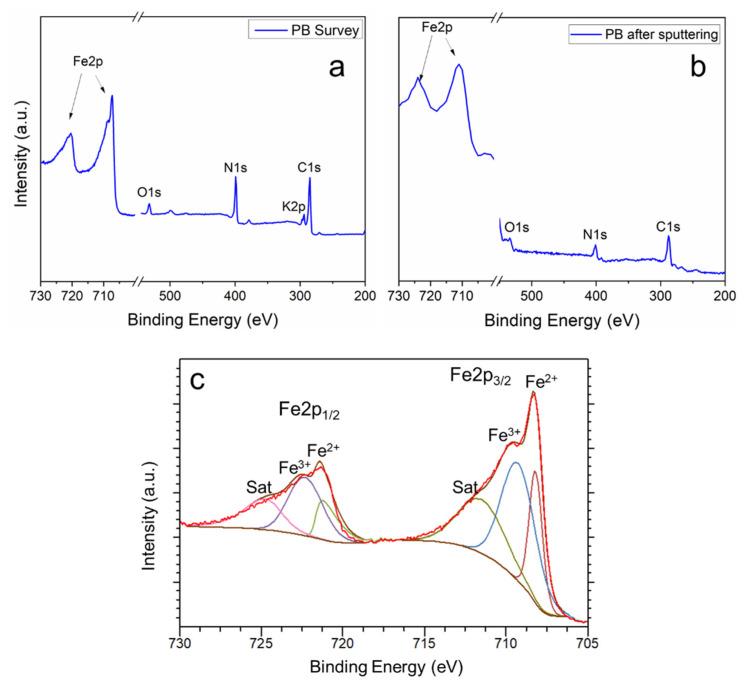
XPS spectra of PB films deposited at 0.3 V and 30 mC of charge: (**a**) survey spectrum of the as-grown film, (**b**) survey spectrum after Ar-ion-sputtering of 20 nm from the surface, (**c**) core level spectrum of Fe 2p measured from the as-grown film.

XPS was used to investigate the elemental composition of the PB thin films. As shown in Figure 4a, the signals of Fe, O, N, K and C can be identified in the spectrum, consistent with the formation of PB. For calibration of the spectra, the C1s peak position 284.6 eV was considered as a reference [31]. The elemental composition at the surface was determined to 14.7 at % Fe, 5.6 at % O, 37.5% N, 5.1 at % K, and 37.2 at % C. The oxygen most probably originates from surface oxides. As expected for CN^−^ ligands, the result indicates a stoichiometric ratio for C and N. A significant amount of K is observed, which can be evidence for sPB, KFe^3+^[Fe^2+^(CN)_6_] in the surface region of the film. On the other hand, the amount of Fe found in our samples is higher than in stoichiometric sPB (~13 at % Fe expected) and lower than in stoichiometric iPB ${\mathrm{Fe}}_{4}^{3+}{\left[{\mathrm{Fe}}^{2+}{\left(\mathrm{CN}\right)}_{6}\right]}_{3}$ (~16 at % Fe expected), indicating that both types of PB could appear in the surface region. In addition, small amounts of the FeCl_3_ and KCl electrolytes on the film surface can be a source, respectively, for Fe and K. To further analyze the composition of the PB film along its section, XPS depth analysis was performed. The XPS survey spectrum after removing 20 nm of the PB film by sputtering is shown in Figure 4b. The spectrum clearly shows the presence of Fe, C, and N as expected for PB. However, no significant amount of K could be identified within the PB film. A similar result was obtained with other sputter depths (not shown). We conclude that preferential sputtering of K occurred since bulk measurement with EDS showed an amount of K of ~3.5 at %, consistent with a mixture of phases.

Figure 4c shows the core level region of Fe 2p and the related curve fits. In the first energy range between 705 and 715 eV, the $\mathrm{Fe}\;2p_{3/2}$ states and in the second energy range between 720 and 730 eV, its respective doublet corresponding to the $\mathrm{Fe}\;2p_{1/2}$ states appear. The fitting results of the peaks demonstrate the coexistence of ${\mathrm{Fe}}^{2+}$ (708.1 eV and 721.3 eV) and ${\mathrm{Fe}}^{3+}$ (709.4 eV and 722.6 eV) and their respective satellites (711.5 eV and 724.7 eV), with a constant energy separation between the Fe 2p states of 13.2 eV [31]. These results are qualitatively consistent with the Raman/SEM measurements and confirm that Fe^3+^ ions were converted during electrodeposition to Fe^2+^ ions, resulting in the formation of iPB.

**Figure 5:**
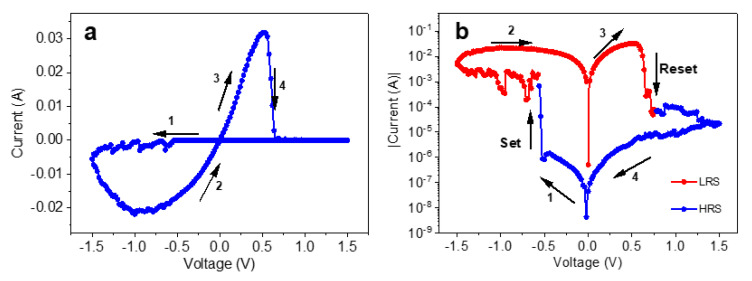
(**a**) I–V curve for the Au/PB/Ag layer stack showing the resistive switching effect. (**b**) Logarithmic plot of absolute current overvoltage. The red and blue part of the curve shows the LRS and HRS regimes, respectively.

In Figure 5a, the result of the electrical measurement for the PB film in a sandwich structure with the layer sequence Au (bottom contact)/PB/Ag (top contact) is shown. We obtain an I–V curve typical for bipolar resistive switching effect [4]. To perform the measurement, a scanning potential is applied with the sequence 0 V→−1.5 V→0 V→+1.5 V→0 V, closing a cycle. The “set” and “reset” switching steps occur in the positive and negative bias regions, respectively. Before applying a voltage, the film remains at high resistance. When applying a bias voltage between 0 and −1.5 V, the current starts to increase at an applied voltage of about −0.6 V and turns the film to a low resistance state (LRS); this action is called “set”. The maximum current reached was −23 mA when the returning voltage was ca. −1.0 V. The film held the LRS until the applied voltage reached a value of ~+0.6 V, inducing the “reset” process, which turned the film back to the high resistive state (HRS). Figure 5b is the same I–V curve in a semi-log scale showing a switching current higher than three orders of magnitude at 0.3 V. The curve clearly shows a butterfly shape that is characteristic of bipolar resistive switching devices [32].

**Figure 6:**
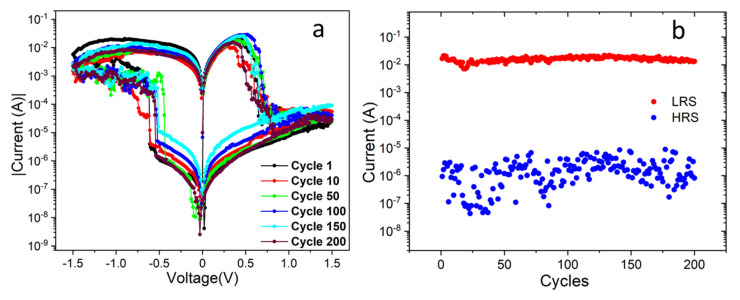
(**a**) I–V curves for Au/PB/Ag samples during 200 cycles. The applied DC bias voltage was set from −1.5 to 1.5 V. (**b**) Switching current of the device between the LRS and HRS at 0.3 V as a function of the number of cycles.

Figure 6a shows the cyclic performance of the Au/PB/Ag device. The current flowing through the device exhibits a similar behavior over several cycles, which confirms the reversibility and reproducibility of the resistive switching mechanism in our samples. Figure 6b displays the current switching at 0.3 V. A ratio of at least three orders of magnitude is observed between LHS and HRS, besides the relatively large scattering for low currents, i.e., for the HRS regime. The HRS of the structures can be read and reprogrammed to the LRS again in the subsequent positive sweeps, thus completing the bipolar “write–read–erase–read–rewrite” cycle for a nonvolatile random-access memory device.

**Figure 7:**
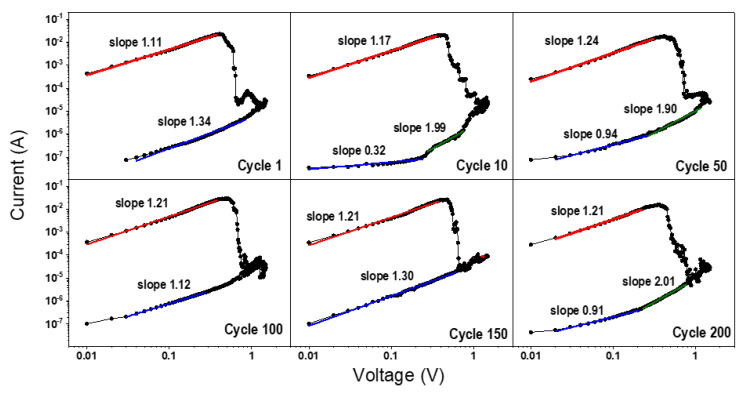
The current–voltage curves replotted on a log–log scale in the positive bias voltage range for cycles 1, 10, 50, 100, 150 and 200.

The conduction behavior of the RS device is studied in the region of positive potentials for the LRS and HRS. The I-V curves for the 1st, 10th, 50th, 100th, 150th and 200th sweeps are plotted in log-log scale in Figure 7 for a sample submitted to 200 cycles. For the LRS, the slopes as close to 1, indicating a linearly ohmic behavior characteristic of conduction through filaments [33,34,35]. The HRS exhibits a less reproducible behavior with the conduction mechanism dependent on specific cycles, i.e., for cycles 1, 100, and 150, slopes of 1.34, 1.12, and 1.30 are observed and could be associated with ohmic conduction. For the cycles 10, 50, and 200, slopes close to 2 are observed for higher voltages (above 0.2 V) that can be described by the SCLC (space charge limited current) model for non-filamentary conduction, similarly to what was observed by Kim and co-authors [34].

**Figure 8:**
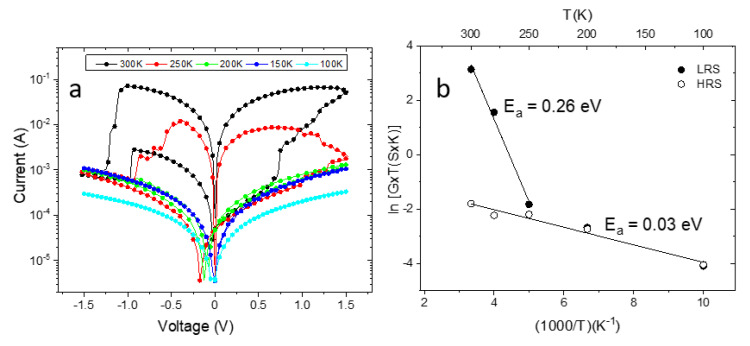
(**a**) Low-temperature dependence of current as a function of voltage for the Ag/PB/Au memory device. (**b**) Dependence of the current at 0.3 V.

To describe the type of conduction that is present in our material, electrical measurements were taken at low temperatures in vacuum. Figure 8a shows the current curves as a function of the potential in a log–log scale for different temperatures measured in our Au/PB/Ag memory structures. We can observe an increase in the measured current with the temperature, as expected. An intermediate level for the HRS is also observed, which is more evident for the sample measured at room temperature (300 K), and could explain the scattering in current shown in Figure 6b. In addition, the temperatures of 100 and 150 K were not sufficient to allow the set/reset process in the range of potentials used.

In Figure 8b, the dependence of the product G·T is presented, where G is the conductance, at 0.3 V in an Arrhenius plot. The results confirm that conduction is a thermally activated process. To determine the activation energy (E_a_) Equation (1) was used,

(1) $$G=\frac{G_{0}}{k_{B}\cdot T}\cdot e^{-\frac{E_{a}}{k_{B}\cdot T}},$$

where G_0_ is a pre-exponential factor and k_B_ the Boltzmann constant, for temperatures of 200, 250, and 300 K, when the device entered in LRS, the activation energy of 0.26 eV was calculated. For HRS, a much lower activation energy of 0.03 eV was obtained. The activation energy for the LHS is similar to the one of 0.27 eV for the conduction in Cu^II^[Fe^III^(CN)_6_]_2/3_·nH_2_O in dry atmosphere obtained by Hosseini et al. [36]. The authors studied the intrinsic conduction in copper hexacyanoferrates and attributed the activation energies to the proton hopping mechanism. Considering the observed low activation energies, they also ruled out the possibility of potassium ions as a possible pathway in the compound KCu^II^[Fe^III^(CN)_6_] due to charge neutrality and the size of the ions.

The ohmic behavior in the LRS state observed in Figure 7 has generally been attributed to conductive filament formation [35]. Since we have observed large grains and grain boundaries crossing the thickness of the electrodeposited layers (see Figure 1 and Figure 2), it is expected that the activation energy of 0.26 eV for the LRS is associated with the formation of channels in these grain boundaries. The HRS activation energy of 0.03 eV could be due to lattices defects (vacancies and interstitials) of the structure of the electrodeposited Prussian blue. Similar values of activation energy of 54 meV for LRS and 74 meV for HRS were reported for the conduction in a Pt/TiO_2_/Pt stack and attributed to the presence of Ti interstitials [34].

## 4. Conclusions

We observed the resistive switching mechanism in electrodeposited layers of Prussian blue. The layers have a homogeneous composition and large grains crossing the full thickness of the electrodeposits. The Au/PB/Ag device presented a bipolar switching of at least three orders in the magnitude of the current, and the effect persisted for 200 cycles, the maximum number of cycles tested. The electrical measurements are consistent with the ohmic conduction model for the low resistance state, and an activation energy of 0.26 eV was calculated for this regime. Future work will give more insight into the relationship between growth conditions, film properties (e.g., thickness, lateral size, composition, morphology), and the underlying conduction mechanism responsible for resistive switching.
